# Supplementary material for: Prediction of five-year mortality after COPD diagnosis using primary care records
Source: PLoS One. 2020 Jul 21;15(7):e0236011. doi: 10.1371/journal.pone.0236011 (PMC7373295; doi:10.1371/journal.pone.0236011)
Supplement: S1 Table — The modelling methods were logistic regression, random forests (a popular machine-learning technique) and Cox regression (i.e. Cox proportional hazards). The variable sets were: just basic variables; basic variables and co-morbidity score; and basic variables and co-morbidity indicators. Logistic regression (glm) and random forest (randomForest) analyse survival as a binary variable: death within five years of COPD diagnosis. Cox regression (survival) analyses survival as a time to event outcome, in this case with survival times censored at 5 years after COPD diagnosis. This censoring has been advocated as a way to improve predictions. Logistic regression and Cox regression were performed with ridge penalisation, lasso penalisation or no penalisation, and, when the variable set included co-morbidity indicators, both with and without pairwise interactions between these indicators. The Aalen-Nelson estimator of the baseline hazard was used to make predictions from the fitted Cox regression model. CRP = C-reactive protein. Default settings were used for all methods and nested cross-validation of penalized models was used to choose the penalty parameter (cv.glmnet). Co-morbidity indicators and pairwise interactions between co-morbidity indicators were only included in relevant models if they were >1% prevalent, e.g. a pairwise interaction between co-morbidities was only included if at least 1% of patients had both. (DOCX) [file pone.0236011.s001.docx]

**S1 Table**

| **Variables** | **Model** | **Taking account of time to event?** | **Penalisation** |
| --- | --- | --- | --- |
| Basic (B) | Cox | Yes | No |
| B | Logistic | No | No |
| B + Cambridge multimorbidity score (CMS) | Cox | Yes | No |
| B + CMS | Logistic | No | No |
| B + Co-morbidities (C) | Cox | Yes | No |
| B + C | Logistic | No | No |
| + diagnosis year | Logistic | No | No |
| + albumin, CRP & platelets | Logistic | No | No |
| + quadratic terms | Logistic | No | No |
| + cubic terms | Logistic | No | No |
| B + C | Cox | Yes | Lasso |
| B + C | Logistic | No | Lasso |
| B + C | Cox | Yes | Ridge |
| B + C | Logistic | No | Ridge |
| B + C + Pairwise co-morbidity interactions (C^2) | Cox | Yes | No |
| B + C + C^2 | Logistic | No | No |
| B + C + C^2 | Cox | Yes | Lasso |
| B + C + C^2 | Logistic | No | Lasso |
| B + C + C^2 | Cox | Yes | Ridge |
| B + C + C^2 | Logistic | No | Ridge |
| All (i.e. non-linear) | Random forest | No | No |
