# Supplementary material for: Prediction of five-year mortality after COPD diagnosis using primary care records
Source: PLoS One. 2020 Jul 21;15(7):e0236011. doi: 10.1371/journal.pone.0236011 (PMC7373295; doi:10.1371/journal.pone.0236011)
Supplement: S2 Table — (DOCX) [file pone.0236011.s002.docx]

**S2 Table**

| **Barnett co-morbidities** | **Prevalence** |
| --- | --- |
| Hypertension | 38% |
| Painful condition | 30% |
| Asthma | 20% |
| Coronary heart disease | 18% |
| Depression | 17% |
| Hearing loss | 15% |
| Diabetes | 11% |
| Thyroid disorders | 8% |
| Anxiety | 8% |
| Stroke and TIA | 7% |
| Diverticular disease | 7% |
| Irritable bowel syndrome | 7% |
| Atrial fibrillation | 7% |
| Prostate disorders | 7% |
| Constipation | 6% |
| Heart failure | 5% |
| Connective tissue disorders | 5% |
| Chronic kidney disease | 5% |
| Peripheral vascular disorder | 5% |
| Cancer | 4% |
| Chronic sinusitis | 4% |
| Alcohol problems | 3% |
| Bronchiectasis | 2% |
| Blindness and low vision | 2% |
| Psoriasis or eczema | 2% |
| Psychosis/bipolar | 2% |
| Substance abuse | 2% |
| Epilepsy | 1% |
| Inflammatory bowel disease | 1% |
| Chronic liver disease | <1% |
| Dementia | <1% |
| Anorexia/bulimia | <1% |
| Parkinson's | <1% |
| Migrane | <1% |
| Multiple sclerosis | <1% |
| Learning disability | <1% |
