# Supplementary material for: Prediction of five-year mortality after COPD diagnosis using primary care records
Source: PLoS One. 2020 Jul 21;15(7):e0236011. doi: 10.1371/journal.pone.0236011 (PMC7373295; doi:10.1371/journal.pone.0236011)
Supplement: S1 Fig — Boxplot showing median and interquartile ranges for (a) prediction accuracy (Brier score), (b) discrimination (AUC = Area Under the Curve) and (c) calibration slope of the prediction models. ‘B’ variables include age, gender, socioeconomic status, smoking status, BMI (value and testing indicator), FEV1% predicted (value and testing indicator). ‘CCI’ is a single variable (derived from 17 variables), the Charlson Co-morbidity Index. ‘CMS’ is a single variable (derived from 20 variables), the general Cambridge Multimorbidity Score, which depends on the presence of Barnett co-morbidities. ‘C’ includes a separate term for each co-morbidity variable. ‘C^2’ includes main effects and pairwise interactions between each co-morbidity variable. ‘All’ includes all basic and co-morbidity variables in a non-linear fashion. For (a) and (b) the red dashed line indicates the best median value over all modelling strategies, whereas for (c) it indicates the perfect calibration (slope = 1). CRP = C-reactive protein. (DOCX) [file pone.0236011.s004.docx]

**a)**

**b)**

**c)**
