# Supplementary material for: Prediction of five-year mortality after COPD diagnosis using primary care records
Source: PLoS One. 2020 Jul 21;15(7):e0236011. doi: 10.1371/journal.pone.0236011 (PMC7373295; doi:10.1371/journal.pone.0236011)
Supplement: S1 File — (DOC) [file pone.0236011.s006.doc]

# ISAC APPLICATION FORM

# PROTOCOLS FOR RESEARCH USING THE CLINICAL PRACTICE RESEARCH DATALINK (CPRD)

| For ISAC use only | | |
| --- | --- | --- |
| Protocol No.  Submission date  (DD/MM/YYYY) | ...........................  ........................... | ***IMPORTANT***  *Please refer to the* ***guidance*** *for ‘****Completing the ISAC application form’*** *found on the CPRD website (*[*www.cprd.com/isac*](http://www.cprd.com/isac)*). If you have any queries, please contact the ISAC Secretariat at* [*isac@cprd.com*](mailto:isac@cprd.com)*.* |

| SECTION A: GENERAL INFORMATION ABOUT THE PROPOSED RESEARCH STUDY | | |
| --- | --- | --- |
| 1. Study Title**^§^** (*Please state the study title below)*   To assess whether landmarking is better than traditional methods for assessing the relationship between changes in lung function (FEV1) over time and mortality in a primary care COPD cohort with multiple co-morbidities  *^§^Please note: This information will be published on the CPRD’s website as part of its transparency policy.* | | |
| 1. **Has any part of this research proposal or a related proposal been previously submitted to ISAC?**   Yes **^*^** No  **If yes, please provide the previous protocol number/s below. Please also state in your current submission how this/these are related or relevant to this study.*  16_186 | | |
| 1. **Has this protocol been peer reviewed by another Committee? (e.g. grant award or ethics committee)**   Yes**^*^** No  **If Yes, please state the name of the reviewing Committee(s) below and provide an outline of the review process and outcome as an Appendix to this protocol :* A summary of this protocol was peer-reviewed and approved by the MRC non-clinical training and career development panel, leading to the awarding of a MRC Career Development Award to Dr Steven Kiddle | | |
| 1. **Type of Study** (please tick all the relevant boxes which apply)  | Adverse Drug Reaction/Drug Safety |  | Drug Effectiveness |  | | --- | --- | --- | --- | | Drug Utilisation |  | Pharmacoeconomics |  | | Disease Epidemiology |  | Post-authorisation Safety |  | | Health care resource utilisation |  | Methodological Research |  | | Health/Public Health Services Research |  | Other^*^ |  |     *^*^If Other, please specify the type of study in the lay summary* | | |
| 1. **Health Outcomes to be Measured^§^**   **^§^***Please note:* *This information will be published on CPRD’s website as part of its transparency policy.*  Please summarise below the primary/secondary health outcomes to be measured in this research protocol:   | - Death |  |  | | --- | --- | --- | |  |  |  | |  |  |  |   [Please add more bullet points as necessary] | | |
| 1. **Publication: This study is intended for** (please tick all the relevant boxes which apply)**:**   Publication in peer-reviewed journals  Presentation at scientific conference  Presentation at company/institutional meetings  Regulatory purposes  Other**^*^**  **If Other, please provide further information:* | | |
| SECTION B: INFORMATION ON INVESTIGATORS AND COLLABORATORS | | |
| 1. **Chief Investigator^§^**   Please state the full name, job title, organisation name & e-mail address for correspondence - see guidance notes for eligibility. Please note that there can only be one Chief Investigator per protocol.  Steven Kiddle, Visiting Research Fellow, Imperial, and Research Fellow, MRC Biostatistics Unit, University of Cambridge, s.kiddle@imperial.ac.uk  **^§^***Please note:* *The name and organisation of the Chief Investigator and will be published on CPRD’s website as part of its transparency policy*  CV has been previously submitted to ISAC  **CV number:** 650_16S  A new CV is being submitted with this protocol  An updated CV is being submitted with this protocol | | |
| 1. **Affiliation of Chief Investigator** (full address)   MRC Biostatistics Unit, Cambridge Institute of Public Health, Forvie Site, Robinson Way, Cambridge Biomedical Campus, Cambridge CB2 0SR, United Kingdom  (Tel) 07850 423332 | | |
| 1. **Corresponding Applicant^§^**   Please state the full name, affiliation(s) and e-mail address below:    **^§^***Please note:* *The name and organisation of the corresponding applicant and their organisation name will be published on CPRD’s website as part of its transparency policy*  Same as chief investigator  CV has been previously submitted to ISAC  **CV number:**  A new CV is being submitted with this protocol  An updated CV is being submitted with this protocol | | |
| 1. **List of all investigators/collaborators^§^**   Please list the full name, affiliation(s) and e-mail address* of all collaborators, other than the Chief Investigator below:  **^§^***Please note: The name of all investigators and their organisations/institutions will be published on CPRD’s website as part of its transparency policy*  Other investigator: Jennifer Quint, Clinical Senior Lecturer in Respiratory Epidemiology, Imperial College London, j.quint@imperial.ac.uk  CV has been previously submitted to ISAC  **CV number:** 042_15CEPSL  A new CV is being submitted with this protocol  An updated CV is being submitted with this protocol  Other investigator: Hannah Whittaker, Imperial College London, h.whittaker@imperial.ac.uk  CV has been previously submitted to ISAC  **CV number:** 535_17  A new CV is being submitted with this protocol  An updated CV is being submitted with this protocol  Other investigator:  CV has been previously submitted to ISAC  **CV number:**  A new CV is being submitted with this protocol  An updated CV is being submitted with this protocol  Other investigator:  CV has been previously submitted to ISAC  **CV number:**  A new CV is being submitted with this protocol  An updated CV is being submitted with this protocol  [Please add more investigators as necessary]  **Please note that your ISAC application form and protocol* ***must*** *be copied to all e-mail addresses listed above at the time of submission of your application to the ISAC mailbox. Failure to do so will result in delays in the processing of your application.* | | |
| 1. **Conflict of interest statement***   Please provide a draft of the conflict (or competing) of interest (COI) statement that you intend to include in any publication which might result from this work  There are no conflicts of interest.  **Please refer to the International Committee of Medical Journal Editors (ICMJE) for guidance on what constitutes a COI.* | | |
| 1. **Experience/expertise available**   Please complete the following questions to indicate the experience/ expertise available within the team of investigators/collaborators actively involved in the proposed research, including the analysis of data and interpretation of results.  **Previous GPRD/CPRD Studies** **Publications using GPRD/CPRD data**  None  1-3  > 3 | | |
| **Experience/Expertise available** | **Yes** | **No** |
| **Is statistical expertise available within the research team?**  *If yes, please indicate the name(s) of the relevant investigator(s)*  Quint, Kiddle |  |  |
| **Is experience of handling large data sets (>1 million records) available within the research team?**  *If yes, please indicate the name(s) of the relevant investigator(s)*  Quint, Whittaker |  |  |
| **Is experience of practising in UK primary care available to or within the research team?**  *If yes, please indicate the name(s) of the relevant investigator(s)*  Quint |  |  |
| 1. **References relating to your study**   Please list up to 3 references (most relevant) relating to your proposed study:   1. Vestbo J, Edwards LD, Scanlon PD, et al. (2011) Changes in forced expiratory volume in 1 second over time in COPD. N Engl J Med. 365(13):1184–1192. *[Shows heterogeneity in FEV1 decline]* 2. Houwelingen et al., (2011) Dynamic Prediction in Clinical Survival Analysis. Chapman & Hall/CRC Monographs on Statistics & Applied Probability. *[Landmark analysis in clinical epidemiology]* 3. Guerra et al., (2018) Large-scale external validation and comparison of prognostic models: an application to chronic obstructive pulmonary disease. BMC Medicine 194 (5) *[Shows existing prognostic models for COPD all-cause mortality]* | | |
| SECTION C: ACCESS TO THE DATA | | |
| 1. **Financial Sponsor of study^§^**   **^§^***Please note:* *The name of the source of funding will be published on CPRD’s website as part of its transparency policy*  Pharmaceutical Industry  *Please specify name and country:*  Academia  *Please specify name and country:* Medical Research Council, UK  Government / NHS  *Please specify name and country:*  Charity *Please specify name and country:*  Other  *Please specify name and country:*  None | | |
| 1. **Type of Institution conducting the research**   Pharmaceutical Industry *Please specify name and country:*  Academia *Please specify name and country:* Imperial College London, UK  Government Department *Please specify name and country:*  Research Service Provider *Please specify name and country:*  NHS *Please specify name and country:*  Other  *Please specify name and country:* | | |
| 1. **Data access arrangements**   The financial sponsor/ collaborator* has a licence for CPRD GOLD and will extract the data  The institution carrying out the analysis has a licence for CPRD GOLD and will extract the data**  A data set will be provided by the CPRD^¥€^  CPRD has been commissioned to extract the data and perform the analyses^€^  Other:  *If Other, please specify:*  **Collaborators supplying data for this study must be named on the protocol as co-applicants.*  ***If data sources other than CPRD GOLD are required, these will be supplied by CPRD*  ^¥^*Please note that datasets provided by CPRD are limited in size; applicants should contact CPRD (*[*kc@cprd.com*](mailto:kc@cprd.com)*) if a dataset of >300,000 patients is required.*  ^€^*Investigators must discuss their request with a member of the CPRD Research team before submitting an ISAC application. Please contact the CPRD Research Team on +44 (20) 3080 6383 or email (*[*kc@cprd.com*](mailto:kc@cprd.com)*) to discuss your requirements. Please also state the name of CPRD Research team with whom you have discussed this request (provide the date of discussion and any relevant reference information):*  Name of CPRD Researcher       Reference number (where available)       Date of contact | | |
| 1. **Primary care data**   Please specify which primary care data set(s) are required)  Vision only (Default for CPRD studies Both Vision and EMIS®*  EMIS® only*    *Note: Vision and EMIS are different practice management systems. CPRD has traditionally collected data from Vision practice. Data collected from EMIS is currently under evaluation prior to wider release.*  **Investigators requiring the use of EMIS data* ***must*** *discuss the study with a member of the CPRD Research team before submitting an ISAC application*  Please state the name of the CPRD Researcher with whom you have discussed your request for EMIS data:  Name of CPRD Researcher       Reference number (where available)       Date of contact | | |
| SECTION D: INFORMATION ON DATA LINKAGES | | |
| 1. **Does this protocol seek access to linked data**   Yes*  No If No, please move to section E.  **Research groups which have not previously accessed CPRD linked data resources* ***must*** *discuss access to these resources with a member of the CPRD Research team, before submitting an ISAC application. Investigators requiring access to HES Accident and Emergency data, HES Diagnostic Imaging Dataset and PROMS data* ***must*** *also discuss this with a member of the CPRD Research team before submitting an ISAC application. Please contact the CPRD Research Team on +44 (20) 3080 6383 or email* [*kc@cprd.com*](mailto:kc@cprd.com) *to discuss your requirements* ***before*** *submitting your application.*  Please state the name of the CPRD Researcher with whom you have discussed your linkage request.  Name of CPRD Researcher: Jenny Campbell Reference number (where available): OCR8666  Date of contact: 22/11/16  *Please note that as part of the ISAC review of linkages, your protocol may be shared - in confidence - with a representative of the requested linked data set(s) and summary details may be shared - in confidence - with the Confidentiality Advisory Group of the Health Research Authority.* | | |
| 1. **Please select the source(s) of linked data being requested^§^**   *^§^Please note: This information will be published on the CPRD’s website as part of its transparency policy.*   | ONS Death Registration Data | MINAP (Myocardial Ischaemia National Audit Project) | | --- | --- | | HES Admitted Patient Care | Cancer Registration Data* | | HES Outpatient | PROMS (Patient Reported Outcomes Measure)** | | HES Accident and Emergency | CPRD Mother Baby Link | | HES Diagnostic Imaging Dataset |  |     Practice Level Index of Multiple Deprivation (Standard)  Practice Level Index of Multiple Deprivation (Bespoke)  Patient Level Index of Multiple Deprivation***  Patient Level Townsend Score ***  Other**** *Please specify:*  **Applicants seeking access to cancer registration data must complete a Cancer Dataset Agreement form (available from CPRD). This should be submitted to the ISAC as an appendix to your protocol.* *Please also note that applicants seeking access to cancer registry data must provide consent for publication of their study title and study institution on the UK Cancer Registry website.*  ***Assessment of the quality of care delivered to NHS patients in England undergoing four procedures: hip replacement, knee replacement, groin hernia and varicose veins. Please note that patient level PROMS data are only accessible by academics*  **** ‘Patient level IMD and Townsend scores will not be supplied for the same study*  *****If “Other” is specified, please provide the name of the individual in the CPRD Research team with whom this linkage has been discussed.*  Name of CPRD Researcher       Reference number (where available)       Date of contact | | |
| 1. **Total number of linked datasets requested including CPRD GOLD**   Number of linked datasets requested *(practice/ ’patient’ level Index of Multiple Deprivation, Townsend Score or the CPRD Mother Baby Link should* ***not*** *be included in this count)*  3  *Please note: Where ≥5 linked datasets are requested, approval may be required from the Confidentiality Advisory Group (CAG) to access these data* | | |
| 1. **Is linkage to a local^¥^ dataset with <1 million patients being requested?**   Yes *  No  **If yes, please provide further details:*  **^¥^** *Data from defined geographical areas i.e. non-national datasets.* | | |
| 1. **If you have requested one or more linked data sets, please indicate whether the Chief Investigator or any of the collaborators listed in question 5 above, have access to these data in a patient identifiable form (e.g. full date of birth, NHS number, patient post code), or associated with an identifiable patient index.**   Yes*  No  ** If yes, please provide further details:* | | |
| 1. **Does this study involve linking to patient *identifiable* data (e.g. hold date of birth, NHS number, patient post code) from other sources?**   Yes  No | | |
| SECTION E: VALIDATION/VERIFICATION | | |
| 1. **Does this protocol describe a purely observational study using CPRD data?**   Yes*  No**  ** Yes: If you will be using data obtained from the CPRD Group, this study does not require separate ethics approval from an NHS Research Ethics Committee.*  *** No: You may need to seek separate ethics approval from an NHS Research Ethics Committee for this study. The ISAC will provide advice on whether this may be needed.* | | |
| 1. **Does this protocol involve requesting any additional information from GPs?**   Yes*  No  * *If yes, please indicate what will be required:*  Completion of questionnaires by the GP*^ψ^* Yes  No  Is the questionnaire a validated instrument? Yes  No  If yes, has permission been obtained to use the instrument? Yes  No  Please provide further information:  Other (please describe)  *^ψ^ Any questionnaire for completion by GPs or other health care professional must be approved by ISAC before circulation for completion.* | | |
| 1. **Does this study require contact with patients in order for them to complete a questionnaire?**   Yes*  No  **Please note that any questionnaire for completion by patients must be approved by ISAC before circulation for completion.* | | |
| 1. **Does this study require contact with patients in order to collect a sample?**   Yes*  No  ** Please state what will be collected:* | | |
| SECTION F: DECLARATION | | |
| 1. **Signature from the Chief Investigator**  - I have read the guidance on ‘***Completion of the ISAC application form****’* and ‘***Contents of CPRD ISAC Research Protocols***’ and have understood these; - I have read the submitted version of this research protocol, including all supporting documents, and confirm that these are accurate. - I am suitably qualified and experienced to perform and/or supervise the research study proposed. - I agree to conduct or supervise the study described in accordance with the relevant, current protocol - I agree to abide by all ethical, legal and scientific guidelines that relate to access and use of CPRD data for research - I understand that the details provided in sections marked with (^§^) in the application form and protocol will be published on the CPRD website in line with CPRD’s transparency policy. - I agree to inform the CPRD of the final outcome of the research study: publication, prolonged delay, completion or termination of the study.   Name: Steven John Kiddle Date: 22/5/17 e-Signature (type name): SJKiddle | | |

**PROTOCOL INFORMATION REQUIRED**

The following sections below **must** be included in the CPRD ISAC research protocol. Please refer to the guidance on ‘***Contents of CPRD ISAC Research Protocols***’ ([www.cprd.com/isac](http://www.cprd.com/isac)) for more information on how to complete the sections below. Pages should be numbered. All abbreviations must be defined on first use.

| **Applicants must complete all sections listed below**  **Sections which do not apply should be completed as ‘*Not Applicable’*** |
| --- |
| 1. **Study Title^§^**   **^§^***Please note:* *This information will be published on CPRD’s website as part of its transparency policy*  To assess whether landmarking and/or latent classes are better than traditional methods for assessing the relationship between changes in lung function (FEV1) over time and mortality in a primary care COPD cohort with multiple co-morbidities |
| 1. **Lay Summary (Max. 200 words)^§^**   **^§^***Please note:* *This information will be published on CPRD’s website as part of its transparency policy*  Lung function tests relate to how well our lungs are working and measure such things as how much air our lungs can hold and how easily we can breathe in and out. All individuals have some decline in their lung function over time, particularly in later life. People with chronic obstructive pulmonary disease (COPD) lose lung function faster than the general population, particularly those who continue to smoke. Lower lung function is associated with premature death and may lead to the inability to perform simple physical tasks such as walking short distances unaided. Studies done have shown great variability in lung function decline of COPD patients, but have not yet related this to what other diseases a patient may have, which are called co-morbidities, or to how long they can expect to live. Therefore, in this project we will study whether an integrated model of co-morbidities, lung function decline and time of death can produce more accurate risk scores for COPD patients. |
| 1. **Technical Summary (Max. 200 words**)**^§^**   **^§^***Please note:* *This information will be published on CPRD’s website as part of its transparency policy*  People with COPD have a faster decline in their lung function than people without COPD. However, little is known about how quickly lung function declines in a primary care cohort in an average COPD patient. We are beginning to address this in an already approved clinical epidemiology project (16_186R). In this methodological study we wish to take this work further to study the impact of co-morbidities, testing the ability of landmarking analysis (i.e. dynamic prediction) to model the relationship between co-morbidities and FEV1 decline and their ability to predict mortality in COPD patients. This will be performed using 10-years worth of FEV1 data. In a secondary analysis we will also study latent class analysis can identify COPD patient subtypes with different prognoses. Preliminary work will establish whether co-morbid patients have different FEV1 decline profiles using mixed effect models, using inverse probability weighting to account for selection bias in those with sufficient longitudinal data.  Analyses and interpretation will be performed with informative presence in mind, for example we will focus on prediction of mortality (which is well recorded) rather than on causal analyses which would have been biased by missing data and residual confounding. |
| 1. **Objectives, Specific Aims and Rationale**   (i) The broad research objectives  To develop better survival predictions for COPD patients in primary care using relevant data contained within their health records.  (ii) The specific aims  To investigate whether landmarking (i.e. dynamic prediction) analysis integrating decline in lung function and co-morbidities (heart failure, asthma, ischemic heart disease and/or depression) leads to more accurate survival predictions for a population based representative cohort of COPD patients over a 10-year time period.  (iii) How achievement of the specific aims will further the research objectives  Using routinely collected lung function data (measured every 15 months in people with COPD as part of QoF from 2004), we will test whether a landmarking model incorporating lung function decline and co-morbidity information improves accuracy of survival predictions. The main benefit of this work will be information on prognosis for COPD patients, and the development of a more accurate survival prediction model. A potential secondary benefit could be the identification of clinically relevant disease subtypes based on FEV1 decline trajectories. |
| 1. **Study Background**   Chronic obstructive pulmonary disease (COPD) is characterized by chronic airflow limitation that is not fully reversible, is progressive and is associated with a range of pathological changes in the lung, significant co-morbidities and extra-pulmonary manifestations (1). It is estimated that 3 million individuals in the UK have COPD however, only 1/3 of them are currently diagnosed. Direct UK health care costs secondary to COPD equate to £805 million. COPD is both preventable and treatable and one of the commonest causes of hospital admission. The prevalence of COPD is increasing globally and it is projected to be not only the third leading cause of death, but also the seventh leading cause of disability adjusted life years (DALYs) lost worldwide by 2030.  COPD is an obstructive lung disease, and people with COPD have a faster decline in their lung function (FEV1) than people without COPD. However, among people with COPD the speed with which lung function is lost varies. Little is known particularly in a primary care cohort about how quickly lung function declines in an average COPD patient (2), let alone how this is affected by the presence of co-morbidities or the relation of this to risk of death (3).  As loss of lung function is often used as an outcome in RCTs, our lack of knowledge of average FEV1 decline makes the interpretation of clinical trials outcomes difficult as it is unclear what would constitute a representative “baseline” rate of loss of lung function. COPD is a very heterogeneous disease, with groups of clinical, pathophysiological and demographic characteristics considered to be important in describing the natural history of disease, and which may be useful in describing distinct phenotypes, targeting therapies or predicting risk. One source of this heterogeneity could be patient co-morbidity. Unfortunately, COPD patients with co-morbidities are typically excluded from clinical trials (4). Better understanding of the impact of co-morbidities on COPD progression will encourage the inclusion of co-morbid patients in COPD trials, and allow point-of-care trials like the Salford Lung Study (5) to be used to identify optimal treatments for co-morbid patients.  Lung function decline (as measured by FEV1) is an important characteristic which may be useful for all three of these purposes. Indeed, previous work has indicated that some patients with COPD have more “active” disease; i.e. lose lung function faster (> 40mls/year) (6,7). While lung function decline is faster in frequent exacerbators (8,9), some COPD patients have more rapid decline in FEV1 irrespective of exacerbations, suggesting that other characteristics seem to be important too. These individuals tend to have mild to moderate disease, be current smokers and have a more emphysematous phenotype. Further work is therefore required to 1) describe the prognosis of patients based on FEV1 decline and co-morbidities; and 2) identify disease subtypes based on FEV1 trajectories that have different prognoses. We believe that this can be addressed through 1) landmarking (i.e. dynamic prediction) survival analysis (13) and 2) latent class analysis (14), which we will assess in this project. |
| 1. **Study Type**   This will be a hypothesis testing study |
| 1. **Study Design**   This will be a retrospective cohort study. |
| 1. **Feasibility counts**   We have undertaken previous work validating spirometry in CPRD in COPD patients (ISAC 12_065A) from 2004 onwards. From other COPD work undertaken in CPRD (using data in individuals eligible for linkage), in a cohort of 220,000 COPD patients, 158, 692 have FEV1 recorded after 2004, 123, 431 have more than one record, 117,321 have more than one record 6 months apart. Of the last group: median follow up for FEV1 records is 3.6 years (IQR 1.9-5.7). 18,803 patients have at least 3 FEV1 measures over at least 3 years. |
| 1. **Sample size considerations**   First we will assess the number of patients with each combination of co-morbidity. Then we will perform power calculations using simulations to determine whether it is realistic to study interactions between different co-morbidities. The most likely outcome will be a joint model of the co-morbidities considered, without interaction terms for simplicity, but if statistical power is sufficient we will consider interaction terms. |
| 1. **Data Linkage Required (if applicable):^§^**   **^§^***Please note that the data linkage/s requested in research protocols will be published by the CPRD as part of its transparency policy*  IMD – to allow use of SES in models  HES – to identify hospitalised exacerbations to determine exacerbation frequency and severity  ONS – to allow use of mortality data in models |
| 1. **Study population**   Patients who have or receive a COPD diagnosis from 2004 onwards. |
| 1. **Selection of comparison group(s) or controls**   Patients with co-morbidities will be compared to those without recorded co-morbidities. Each co-morbidity will be represented by a co-variate (present or absent), so that patients with multiple co-morbidities will be readily incorporated. |
| 1. **Exposures, Health Outcomes^§^ and Covariates**   **^§^***Please note:* *Summary information on health outcomes (as included on the ISAC application form above )will be published on CPRD’s website as part of its transparency policy*  **Exposure:** The primary exposure of interest is a COPD diagnosis. The COPD codelist is published (10).  **Covariates**  Other co-morbid conditions: ischemic heart disease, heart failure, depression and asthma (we have validated asthma recording in ISAC 15_257).  FEV1 (time varying covariate) as determined by spirometry values. This is obtained from the Additional file. Where more than one record is available on the same day, we will use the highest value.  *(Below are additional co-variates that might be included in the models. This list will be refined in our clinical study 16_186R using cross-validation, prior to this work.)*  Demographic: Age, sex, ethnicity, SES, BMI (weight in kilograms divided by height in meters squared), and smoking status (current smoker, ex-smoker, never smoker). These are all recorded in the Clinical/Additional files in the CPRD extract and the group have extensive experience in defining them.  COPD medications: These will include inhaled corticosteroids, combination inhaled corticosteroids and long acting beta agonists, long and short acting beta agonists, oral prednisolone, short and long acting anti-cholinergics, theophyllines, combination short acting beta agonists and short acting anti-cholinergics, nebulised therapy.  COPD severity: CAT, mMRC, presence of emphysema (we are aware this may not be well coded and are investigating this as part of other work (ISAC 16_103R2), after first hospitalisation for AECOPD (ISAC 13_116RA), eosinophil level, neutrophil level.  **Outcome:** Year of death as determined from ONS. |
| 1. **Data/ Statistical Analysis**   Prior work: The final set of additional co-variates to include in models will be finalised on the basis of cross-validation in the approved clinical study (16_186R), for more details see above.  Analyses and data management will be performed in STATA and R.   1. A descriptive analysis of the data will be performed: (a) providing counts of COPD patients with each type and combination of co-morbidities, (b) a description of the distribution of FEV1 observations, (c) a description of the differences between all COPD patients and the subgroup with at least 3 time points over at least 3 years, and (d) the relationship between FEV1 and rate of change in FEV1 (from a linear model) will be modelled. We will also perform power calculations to inform the other analyses, using simulations to determine whether it is realistic to study interactions between different co-morbidities. The most likely outcome will be a joint model of the co-morbidities considered, without interaction terms for simplicity, but if statistical power is sufficient we will consider interaction terms. 2. A preliminary analysis will use mixed effect models to examine whether co-morbid patients have different FEV1 decline trajectories, i.e. using co-morbidities present at first FEV1 observation as a model co-variate. This analysis will be restricted to patients with at least 3 time points over at least 3 years as recommended by the FDA (11), using inverse probability weighting to account for selection bias. 3. A primary analysis will be performed with landmarking (i.e. dynamic prediction) to use FEV1 decline parameters and co-morbidity present by ‘landmark time’ to predict survival (12). To assess robustness, this analysis will be performed twice and the results compared: (a) restricted to patients with at least 3 timepoints over at least 3 years (11) using inverse probability weighting to account for selection bias, and (b) on all patients, with decline co-efficients set to their average values for patients with insufficient longitudinal data. In the second analysis, length and frequency of follow-up will be included as co-variates, as these could themselves be predictive of mortality. 4. A secondary analysis will perform clustering using a latent class mixture model (13) on patients with at least 3 FEV1 measures over 3 years. Patient subgroups (i.e. latent classes) will then be related to co-morbidites present at first FEV1 observation and prognosis in a survival model. |
| 1. **Plan for addressing confounding**   Potential confounders will be considered as co-variates in models, as indicated. Inverse probability weighting will be used to address selection bias in availability of longitudinal data. However, due to the presence of residual confounding we will focus on predictive ability of recorded data rather than on causal interpretations. |
| 1. **Plans for addressing missing data**   Potential confounding between presence of longitudinal FEV1, co-morbidities and mortality risk will be examined by inverse probability weighting and the inclusion of follow-up length and frequency as co-variates in survival models. Similarly, selection bias due to missing linked or demographic data will be characterised and accounted for in the same manner. |
| 1. **Patient or user group involvement (if applicable)**   We have not discussed this proposal with patients directly. Currently there is no plan to involve patients in the study, but we will involve patients if appropriate. |
| 1. **Plans for disseminating and communicating study results, including the presence or absence of any restrictions on the extent and timing of publication**   The study findings will be submitted for publication in peer-reviewed scientific journals, and will be presented at appropriate conferences and other meetings; the latter will include scientific meetings externally, for example the American and European Respiratory Society Meetings and internally within Imperial College London. We plan to share our findings with patient/user groups via Breathe Easy (part of the British Lung Foundation). |
| 1. **Limitations of the study design, data sources, and analytic methods**   **Selection bias**  The generalizability of this work relies on a representativeness of COPD patients present in CPRD. GP practices are self-selecting into CPRD, and as such there is a potential for selection bias. However, the prevalence and severity of diagnosed COPD in CPRD is broadly similar to expected levels.  In some of our models we will be restricting to patients with at least 3 FEV1 measures over at least 3 years, as recommended by the FDA (11). We will use inverse probability weighting to increase the generalisability of these results, but will also be cautious in causal interpretations due to residual confounding.  **Misclassification**  It is possible that patients may be misclassified due to undiagnosed comorbidities in those with COPD. We will discuss the potential implications of this in any report.  **Data sources**  Only approximately 50% of the time is it stated that spirometry is done post bronchodilator. NICE guidance states that this is necessary for a diagnosis of COPD. We will carry out a sensitivity analysis by repeating the main analysis in both those in whom it is not stated as well as in those in whom it is clearly stated. We will assume that subsequent readings after diagnosis are post bronchodilator if patients are prescribed long acting bronchodilator inhalers. |
| 1. **References**   1. Vestbo J, Hurd SS, Agustí AG, Jones PW, Vogelmeier C, Anzueto A,et al. Global strategy for the diagnosis, management, and prevention of chronic obstructive pulmonary disease: GOLD executive summary. Am J Respir Crit Care Med. 2013; 187:347–65. doi: 10.1164/rccm.201204-0596PP PMID: 22878278  2. Kim SJ, Lee J, Park YS et al. Age related annual decline of lung function in patients with COPD. International Journal of COPD 2016:11 51–60  3. Stallberg B, Teixeira P, Blom C, et al. The prevalence of comorbidities in COPD patients, and their impact on health status and COPD symptoms in primary care patients: a protocol for an UNLOCK study from the IPCRG. npj Primary Care Respiratory Medicine (2016) 26  4. Herland K, Akselsen JP, Skjønsberg OH, Bjermer L. How representative are clinical study patients with asthma or COPD for a larger “real life” population of patients with obstructive lung disease? Respir Med 2005;99:11-9.  5.Vestbo J et al. (2016) Effectiveness of Fluticasone Furoate-Vilanterol for COPD in Clinical Practice. N Engl J Med 75(13):1253-60  6. Vestbo J, Edwards LD, Scanlon PD, Yates JC, Agusti A, Bakke P, et al. Changes in forced expiratory volume in 1 second over time in COPD. N Engl J Med. 2011 Sep 29; 365(13):1184–92. doi: 10.1056/NEJMoa1105482PMID: 21991892  7. Casanova C, de Torres JP, Aguirre-Jaíme A, Pinto-Plata V, Marin JM, Cordoba E et al. The progression of chronic obstructive pulmonary disease is heterogeneous: the experience of the BODE cohort. Am J Respir Crit Care Med. 2011; 184:1015–21. doi: 10.1164/rccm.201105-0831OC PMID: 21836135  8. Donaldson GC et al. Thorax 2002;57:847-852.  9. Kanner RE et al. Am J Respir Crit Care Med 2001;164:358-364.  10. Quint JK, Müllerova H, DiSantostefano RL, Forbes H, Eaton S, Hurst JR, & Smeeth L (2014). Validation of chronic obstructive pulmonary disease recording in the Clinical Practice Research Datalink (CPRD-GOLD).BMJ open, 4(7), e005540.  11. Food and Drug Association, 2016. Chronic Obstructive Pulmonary Disease: Developing Drugs for Treatment Guidance for Industry. http://www.fda.gov/downloads/drugs/guidancecomplianceregulatoryinformation/guidanc es/ucm071575.pdf.  12. Houwelingen et al., (2011) Dynamic Prediction in Clinical Survival Analysis. Chapman & Hall/CRC Monographs on Statistics & Applied Probability.  13. Berry et al., (2016) A Distinct Low Lung Function Trajectory from Childhood to the Fourth Decade of Life. Am J Respir Crit Care Med 194 (5) |
| **List of Appendices** *(Submit all appendices as separate documents to this application)* |

**AMENDMENT**

The following additional protocol amendment is proposed, as of 1/6/2018:

Title

We propose to change the title to:

‘To assess whether landmarking is better than traditional methods for assessing the relationship between changes in lung function (FEV1) over time and mortality in a primary care COPD cohort with multiple co-morbidities’

To reflect the fact that the study of heterogeneity in FEV1 decline is not the focus of the present project. The idea is to focus the methodology study on prognosis.

Investigators

The research team would like to replace Kieran Rothnie with Hannah Whittaker.

Objectives, specific aims and rationale

We propose to look at co-morbidities more broadly, using the list of co-morbidities developed for the seminal study by Barnett et al., (2012) Lancet. We will now longer look for clinically relevant subtypes based on FEV1 as part of this study, given the stronger focus on prognosis.

Sample size considerations

The focus of this study is now on all-cause mortality prediction, rather than on inference, i.e. focusing on survival models, not on models of FEV1 decline. Therefore, we will focus on variable selection and rigorous cross-validation rather than power calculations. For details, see statistical analysis section of amendment.

Study population

We will now study only individuals who receive a COPD diagnosis from 2004 onwards, i.e. incident cases. This is because time from first diagnosis of COPD will be used in survival analysis.

Exposures, health outcomes and covariates

We will now look at 40 common co-morbidities, with codelists available at <http://www.phpc.cam.ac.uk/pcu/cprd_cam/codelists/>. The exceptions are COPD and asthma, for which we will use Quint group definitions which has been validated as part of (10) and ISAC 15_257. We will only include co-morbidities whose prevalence of >1% in models.

Statistical Analysis

Preliminary analysis of factors affecting FEV1 decline and the secondary analysis (latent class mixture models) will now be dropped. Because of this we also will drop descriptive analyses (c) and (d). Power calculation will now be dropped in favour of variable selection and rigorous cross-validation as detailed below.

Cross-validation will be done on the level of practices, to assess generalisability to new practices, and will take two forms. 80% of practices will be used as a training set, within which 10-fold cross-validation will be used to decide on the final model. The held out 20% of practices (the test/validation set) will be used to report the predictive ability of this final model. Variable selection will be performed in the training set using penalized parametric survival analysis, with penalties selected based on the 10-fold cross-validation. This gives us freedom to explore higher-level interactions, with those not useful for prediction excluded from the model.

Landmarking models that include longitudinal FEV1 decline data will be compared with survival models that use just cross-sectional FEV1 data, and complex co-morbidity models will be compared with simpler methods including the charlson co-morbidity index and COPD specific scores such as those compared in Guerra et al., (2018) BMC Medicine. We may also compare these approach to logistic regression or classification models based on survival to 5-years and 10-years post-diagnosis.

Plan for addressing confounding and missing data

We will no longer use inverse probability weighting, as we will focus on prediction of mortality (which is uniformly well recorded). As previously indicated, we will include missingness and number of observation (e.g. for FEV1) indicators in the prediction models, as these may be proxy variables for other factors (such as general health) which may be useful for prediction.
